# Multicomponent exercise intervention impact on recovery outcomes in frail older adult patients with trochanteric femoral fractures: a quasi-experimental study

**DOI:** 10.3389/fmed.2026.1993562

**Published:** 2026-10-29

**Authors:** Hua Lin, Yang Wang, Yali He, Ying Zhu

**Affiliations:** Department of Nursing, Honghui Hospital, Xi'an Jiaotong University, Xi'an, Shaanxi, China

**Keywords:** frailty, intramedullary, multicomponent exercise, older adults, trochanteric femoral fractures

## Abstract

**Purpose:**

This study investigated Trochanteric femoral fractures (TFFs) in frail older adult patients who are at high risk of complications and increased mortality, thereby imposing a significant economic burden on healthcare systems. The objective of this study was to evaluate the efficacy of a multicomponent exercise intervention compared with conventional care in promoting recovery outcomes in older adult patients with frailty post-TFF surgery.

**Patients and methods:**

In this quasi-experimental study with a historical (non-concurrent) control group, we analyzed 120 frail patients with TFFs who were treated with intramedullary nailing between January 2023 and December 2024. Patients were divided into a control group receiving conventional care and an observation group receiving multicomponent exercise interventions, both receiving interventions for 3 months. The multicomponent intervention comprised strength, balance, flexibility, and aerobic exercises, was delivered by a multidisciplinary team, and was progressed from bed-based to standing and walking training. The modified Barthel index (MBI) and Functional Independence Measure (FIM) tools were used to evaluate the rehabilitation status of the two patient groups. The Harris Hip Score (HHS) was used as a secondary outcome to assess hip function. The Clinical Frailty Scale (CFS) score was used to evaluate frailty. The 36-item Short-Form Health Survey (SF-36) was used to assess quality of life.

**Results:**

After the intervention, the MBI, FIM, and SF-36 scores of the observation group were statistically significantly higher than those of the control group (*P* < 0.05). The CFS was significantly lower in the observation group than in the control group (*P* < 0.05). The HHS score was significantly higher in the observation group than in the control group (*P* < 0.05).

**Conclusion:**

These findings suggest the potential benefits of multicomponent exercise interventions in enhancing rehabilitation outcomes and reducing frailty in older adult patients with TFFs, indicating a promising direction for future clinical practice. Future studies should explore the scalability of these interventions across diverse settings and populations to refine and validate the therapeutic strategies identified in this study.

## Introduction

Trochanteric femoral fractures (TFFs) represent a significant public health concern, particularly in older adults. As the global demographic landscape shifts towards an aging society, the incidence of TFFs has increased sharply. Studies indicate that individuals aged >60 years are at a heightened risk, with rates anticipated to rise notably in the coming years owing to factors such as increased life expectancy and the prevalence of osteoporosis among older adults (1). The implications of these fractures extend beyond immediate physical trauma; they are associated with severe functional decline, heightened healthcare costs, and diminished quality of life, thereby exerting substantial pressure on healthcare systems and communities (1–3).

Current treatment options, including surgical intervention and subsequent rehabilitation, frequently yield limited success, characterized by delayed recovery and suboptimal functional outcomes among frail older adults (4). At the same time, due to aging and physical function decline, older adults often have frailty, which brings great difficulties to their rehabilitation after bone fractures and daily life (5). Given the increasing prevalence of frailty in older adults, it is imperative to explore enhanced recovery strategies that specifically address the needs of this vulnerable demographic (6). Traditional rehabilitation care focuses more on postoperative pain and complication prevention for bone fractures, lacking attention to the overall function of older adults (7). Existing literature highlights the role of exercise in improving recovery outcomes for older adults following fractures, particularly in the context of TFFs (8).

Multicomponent exercise interventions include strength training, flexibility training, balance training, and aerobic exercise, aiming to comprehensively improve patients' physical functions through integrated training methods (9). In recent years, it has been widely applied in the field of medical care. Research shows that multicomponent exercise interventions can enhance patients' muscle strength and improve cardiopulmonary function, with potential value in enhancing patients' recovery and quality of life (9, 10). However, there remains a notable gap in research specifically focused on the efficacy of multicomponent exercise interventions in improving rehabilitation outcomes in frail older adults with TFFs.

The first 3 months after a TFF are a particularly critical period for recovery: this window coincides with the early phase of fracture healing and callus remodeling, during which appropriately graded mechanical loading promotes bone regeneration; it is also the period of the most rapid loss of muscle strength, balance, and functional independence when mobilization is delayed, and the functional gains achieved during this early period are largely decisive for long-term recovery.

This study aimed to evaluate the efficacy of a multicomponent exercise intervention compared to traditional care in promoting recovery outcomes, hip function, frailty status, and quality of life during the first 3 months after surgery in older adults with frailty following TFF surgery. By addressing these critical areas, this study aimed to provide a foundation for future research and clinical practices that prioritize the needs of this vulnerable population, ultimately enhancing their quality of life and reducing the burden associated with TFFs and frailty.

## Material and methods

### General information

The inclusion criteria included: (1) recent unilateral TFFs, (2) age ≥60 years, (3) patients exhibiting symptoms of frailty, and (4) time from injury to surgery <2 weeks. The exclusion criteria were as follows: (1) patients with tumors, thyroid disorders, mental illnesses, severe cardiovascular and cerebrovascular diseases, or other major health issues; (2) pathological fractures; (3) coexisting coagulation disorders, lower limb varicose veins, or hematological diseases; (4) coexisting injuries to other vital organs or fractures in other locations; and (5) incomplete medical records, including incomplete follow-up assessment data. We included 120 patients with TFFs (53 males and 67 females) between January 2023 and December 2024 from Xi'an Honghui Hospital. According to the AO/OTA classification, the included fractures comprised type 31-A1 (*n* = 50), type 31-A2 (*n* = 70) patterns, which collectively correspond to trochanteric femoral fractures. Accordingly, the terminology used throughout this manuscript follows the AO/OTA classification of trochanteric femoral fractures. Study design: this was a quasi-experimental study with a historical (non-concurrent) control group: Patients were allocated according to treatment period control group: patients treated between January 2023 and December 2023, who received conventional postoperative care; observation group: patients treated between January 2024 and December 2024, who received the multicomponent exercise intervention as part of a hospital-wide quality improvement initiative. Sixty patients received conventional care after surgery (control group), while 60 patients received a multicomponent exercise intervention postoperatively (observation group). Comparison of the general data between the two groups showed no statistically significant differences (*P* > 0.05), indicating that the groups were comparable (Table 1). No *a priori* sample-size calculation was performed; the sample size was determined by the number of consecutive eligible patients treated during the two predefined calendar-year periods (January–December 2023 for the control group and January–December 2024 for the observation group). The 3-month intervention and follow-up period was chosen because it covers the critical early healing and functional-recovery window after TFF, corresponds to the routine 1- and 3-month postoperative reassessment time points used in our institution, and maximizes the completeness of follow-up in this frail older population; longer-term maintenance of the benefits is acknowledged as an area requiring further study. Our institution routinely collects frailty assessments as part of geriatric orthopedic care, and these data were systematically recorded in the medical records. All data were extracted from a prospectively maintained electronic medical record system at Xi'an Honghui Hospital. Both the patients and their families provided informed consent for participation in the study. This study was approved by the Ethics Committee of Xi'an Honghui Hospital (No. 202508008), and the whole research process followed the relevant requirements of the Helsinki Declaration.

**Table 1:** Comparison of general data between the two groups.

Characteristics	Control group	Observation group	*P*
	(*n* = 60)	(*n* = 60)	
Age, y	71.64 ± 5.63	71.18 ± 4.92	0.634
Sex, *n*			0.713
Male	28	25	
Female	32	35	
BMI (kg/m^2^)	25.8 ± 3.5	24.9 ± 4.1	0.198
Time from injury to surgery			0.851
<48 h	24	22	
≥48 h	36	38	
Causes of fractures			0.895
Fall	50	48	
Traffic accident	5	6	
Other	5	6	

All fractures were stabilized with PFNA-II. Short nails were used, and a single device type were used during the study period. Distal locking screws were routinely employed in static locking mode. Because both groups were operated on by the same surgical teams at the same institution during consecutive years, implant type and locking strategy were identical between the two groups.

Fracture reduction quality was assessed on postoperative anteroposterior and lateral radiographs according to Baumgaertner-Fogagnolo's criteria by two orthopedic surgeons (11). Reduction quality was graded as good, acceptable, or poor (good: *n* = 80; acceptable: *n* = 30; poor: *n* = 10). No patient in either group experienced loss of reduction or fixation failure during the study period.

### Intervention methods

#### Control group intervention method

Conventional nursing care was used. After admission, we provided health education to enhance patients' understanding of their disease and surgery, and offered psychological support to help patients with their emotions and maintain a good mental state. Postoperatively, we guided patients to start early rehabilitation training, encouraging them to get out of bed as soon as possible, moving gradually from the bed to the bedside and then to the floor, slowly increasing how much they do and cutting down on bed rest. For patients in pain, we managed their pain and gave them painkillers as advised by their doctors. Regular follow-ups were performed after the patients were discharged.

#### Observation group intervention method

Based on the control group, we implemented a multicomponent exercise intervention. We formed a team of orthopedic doctors, nurses, physical therapists, and family members. Everyone on the team received extra training using the multi-component exercise approach. Regarding the specific content of the exercise training, we followed the idea of starting easy and moving to harder exercises, gradually increasing the difficulty and intensity of training so that we did not push them too hard. Further details are provided in Table 2. In terms of psychological support, we actively talked to the patients, encouraged them, and boosted their confidence and enthusiasm for the rehabilitation exercises. Methods such as deep breathing and relaxation training were used to help patients alleviate their tension and maintain a calm mindset. Finally, regarding family support, we ensured that family members could assist patients with muscle relaxation training, such as massage and hot compresses, and that they could conduct joint range of motion training, including passive and active-assisted joint movements, to help their joints gradually return to normal. Exercise intensity was individualized according to each patient's tolerance, following the principle of “no pain beyond acceptable fatigue”. Exercises were modified (reduced in intensity or volume) or temporarily discontinued when a patient experienced exercise-aggravated pain (numerical rating scale >4), dizziness, chest tightness or dyspnea, excessive fatigue, or any suspected new injury, and were resumed after reassessment by the responsible physical therapist and the treating physician. The detailed components, frequencies, session durations, executors, intensity, and progression of the program, contrasted with conventional care, are presented in Table 2.

**Table 2:** Specific content of multicomponent exercise intervention for patients in the observation group.

Timing of intervention	Content	Frequency	Executor
1–2 weeks after surgery	Passive exercises in bed (joint mobility exercises) (ankle pumps, quadriceps isometric contractions, and passive hip–knee range-of-motion exercises within the pain-free range)	2 times/day	Nurse
	Breathing training (deep-breathing and effective coughing exercises to prevent pulmonary complications)		Physiotherapist
3–4 weeks after surgery	Muscle-strengthening exercises (isometric and elastic-band resistance exercises; 2–3 sets of 8–12 repetitions per muscle group)	3 times/day	Physiotherapist
	Sit-up exercises (bed-exit and transfer training)		Nurse
5–8 weeks after surgery	Standing exercise, Balancing board practice (progressive partial-to-full weight bearing and balance-board training)	2 times/day	Physiotherapist
	Walking practice with an ambulation aid (walker progressing to a cane as tolerated)		
9–12 weeks after surgery	Stair exercises, strength training (progressive resistance training of 3–4 lower-limb exercises at 60%–80% of one-repetition maximum)	5 times/week	Physiotherapist
	Daily living skills training (e.g., dressing, washing) (task-specific training to restore independence in basic activities of daily living)		Patients’ relatives
Session duration	20–30 min per session in weeks 1–4, increased to 30–45 min per session from week 5 onward	1 times/day	Nurses
Exercise intensity	Individualized at a moderate intensity, with resistance loads progressing from approximately 50% toward 60%–80% of one-repetition maximum; principle of “no pain beyond acceptable fatigue”	/	Physiotherapist
Progression	Bed-based → /transfer → standing/balance → ambulation → stairs and strengthening, according to tolerance	Encouraged to progress activity as tolerated	Physiotherapist
Criteria to modify/discontinue	Pre-specified criteria (see Materials and Methods)	—	Physiotherapist and Orthopedic doctors

The exercise prescription (content, session duration, intensity, and progression) was designed with reference to published multicomponent exercise and progressive resistance training programs for older adults after hip fracture surgery (10, 25, 33).

### Quality control methods

We regularly conducted functional assessments (such as muscle strength tests, balance skills assessments, and activities of daily living assessments) to monitor rehabilitation progress and make timely adjustments to the intervention plans. Detailed records were kept of the time, content, patient responses, and any abnormalities for each intervention that provide a basis for future analysis and improvements. Regular multidisciplinary team meetings were held to work together on issues that arose during rehabilitation. We also strengthened health education for patients and their families to help them understand the importance of rehabilitation and boost their self-management skills.

Adherence to the exercise program and participant safety were monitored throughout the study. For every prescribed session, the responsible team member recorded in an individual rehabilitation log whether the session was completed. Any withdrawal or non-cooperation was documented together with its reason. All adverse events occurring during or within 24 h after exercise sessions, including falls, fracture-related pain aggravation, reinjury, and cardiovascular symptoms, were recorded in the medical records and reviewed at the regular multidisciplinary team meetings.

### Observation indicators

The MBI and FIM were pre-specified as the primary functional outcome measures of this study. The modified Barthel Index (MBI) (12) and Functional Independence Measure (FIM) (13) were used to evaluate the rehabilitation status of the two patient groups before and after the intervention. The MBI includes 10 items related to daily living activities such as eating, bathing, grooming, dressing, bowel control, bladder control, toileting, transferring between the bed and a chair, walking on level ground, and climbing stairs. Each activity was scored based on the patient's independence, with a total score ranging from 0 to 100. The FIM was developed by the American Congress of Rehabilitation Medicine in 1987 and covers six areas: self-care, sphincter control, transfer, walking/wheelchair use, communication, and social cognition (13). Each item is scored from 1 (totally dependent) to 7 (totally independent), with a maximum score of 126. Higher MBI and FIM scores indicate that patients are better at performing activities of daily living.

The Harris Hip Score (HHS) (14) was used as a secondary, exploratory outcome to assess the patients' hip joint function before the intervention and again 1 and 3 months after; it was retained primarily to permit comparability with previous studies of trochanteric fractures. The HHS includes four dimensions: pain (44 points), function (47 points), range of motion (5 points), and deformity (4 points). Specific items included how much pain they felt, their walking ability, climbing stairs, sitting, putting on socks, and other daily activities, as well as measurements of joint range of motion and the presence of deformities. The maximum score on the HHS is 100, with higher scores indicating better hip joint function.

The Clinical Frailty Scale (CFS) score (15, 16) was used to evaluate the frailty status of patients. The CFS is a nine-point scoring system designed to classify patients based on their functional status and to predict a broad spectrum of functional impairments that contribute to frailty. The scale employs a graded classification as follows: “coping well” (CFS 1–2), “vulnerable” (CFS 3–4), “mildly frail” (CFS 5), “moderately frail” (CFS 6–7), “severely frail” (CFS 8), and an additional category for patients with “terminally illness” status (CFS 9).

The Short Form Health Survey (SF-36) (17) was created to comprehensively assess the patients' quality of life. The SF-36 includes 36 items across eight areas: physical functioning (10 items), physical role functioning (4 items), bodily pain (2 items), general health (5 items), vitality (4 items), social functioning (2 items), emotional role functioning (3 items), and mental health (5 items). The total SF-36 score is 100, with higher scores indicating a better quality of life.

### Statistical analyses

Data were analyzed using SPSS version 27.0. Continuous variables were examined for normality using the Shapiro–Wilk test. Normally distributed measurement data are expressed as means ± standard deviations ($\bar{x}\pm s$); between-group comparisons at each time point were conducted using independent-samples *t*-tests, and within-group pre- to post-intervention comparisons were performed using paired-samples *t*-tests for each group separately. Count data are expressed as frequencies and percentages (%), and comparisons between groups were conducted using the chi-squared test. All *P*-values <0.05 indicated a statistically significant difference. Patients with incomplete medical records, including incomplete follow-up assessment data, were excluded at patient selection (exclusion criterion 5). Consequently, outcome data were complete for all 120 patients at all assessment time points, and no imputation of missing data was required.

## Results

Comparison of the general data between the two groups showed no statistically significant differences (*P* > 0.05), indicating that the groups were comparable (Table 1).

### Intervention adherence and adverse events

Complete rehabilitation logs were available for all patients in the observation group, as patients with incomplete medical records had been excluded at selection. The overall adherence rate, defined as the number of completed sessions divided by the number of prescribed sessions, was 96.8%. No patient withdrew from the program; the less-than-complete adherence was attributable to a small number of patients independently reducing their training volume because of post-exercise pain. During the 3-month intervention period, five adverse events were recorded: one case of poor wound healing, which healed uneventfully after routine wound dressing changes, and four cases of severe pain induced by the exercise sessions, which subsided gradually after rest. No falls, reinjuries, deaths, or reoperations occurred during the study period.

### Rehabilitation outcomes

After intervention, the mean MBI of the patients was statistically higher in the observation group than in the control group (83.62 ± 7.83 vs. 75.12 ± 7.46, respectively; *P* < 0.001). The mean FIM of the patients was found to be significantly higher in the observation group than in the control group (94.84 ± 11.32 vs. 86.03 ± 10.81, respectively, *P* < 0.001). Within both groups, MBI and FIM scores improved significantly from baseline to 3 months after intervention (all *P* < 0.001; Table 3).

**Table 3:** Comparison of MBI, FIM, HHS, CFS, and SF-36 scores between the two groups.

Subject	Control group	Observation group	*P*
	(*n* = 60)	(*n* = 60)	
MBI			
Before intervention	48.74 ± 6.11	49.18 ± 5.76	0.685
After intervention	75.12 ± 7.46	83.62 ± 7.83	<0.001
P	<0.001	<0.001	
FIM			
Before intervention	62.91 ± 7.27	63.58 ± 6.91	0.605
After intervention	86.03 ± 10.81	94.84 ± 11.32	<0.001
P	<0.001	<0.001	
HHS			
Before intervention	32.41 ± 3.41	31.46 ± 3.21	0.118
1 month after intervention	58.59 ± 4.48	66.11 ± 3.92	<0.001
3 months after intervention	82.69 ± 6.31	89.38 ± 5.89	<0.001
P	<0.001	<0.001	
CFS			
Before intervention	7.03 ± 0.61	6.93 ± 0.56	0.351
After intervention	3.52 ± 0.53	2.49 ± 0.44	<0.001
P	<0.001	<0.001	
SF-36			
Before intervention	55.43 ± 4.65	54.88 ± 3.98	0.487
After intervention	79.05 ± 6.43	89.96 ± 5.62	<0.001
P	<0.001	<0.001	

Data are presented as mean ± standard deviation. In each panel, the row labeled “P” indicates the within-group comparison (baseline vs. post-intervention time points) for each group, whereas the *P* values in the rightmost column indicate between-group comparisons at each time point. *P* < 0.05 was considered statistically significant. Between-group comparisons were performed using independent-samples *t*-tests for continuous variables and chi-squared tests for categorical variables; within-group pre- versus post-intervention comparisons were performed using paired-samples *t*-tests.

MBI, modified Barthel Index; FIM, Functional Independence Measure; HHS, Harris Hip Score; CFS, Clinical Frailty Scale; SF-36, 36-item Short Form Health Survey.

### Hip joint function

The average HHS of the observation group patients at 1 and 3 months after intervention was 66.11 ± 3.92 and 89.38 ± 5.89, respectively. The average HHS of the control group patients at 1 and 3 months after intervention was 58.59 ± 4.48 and 82.69 ± 6.31, respectively. At 1 and 3 months after the intervention, the HHS of the observation group was higher than that of the control group (*P* < 0.001). Within both groups, HHS scores improved significantly from baseline to 1 and 3 months after intervention (all *P* < 0.001; Table 3).

### Frailty and quality of life

After intervention, the mean CFS score of the patients was significantly lower in the observation group than in the control group (2.49 ± 0.44 vs. 3.52 ± 0.53, respectively; *P* < 0.001). Within both groups, CFS scores decreased significantly from baseline to 3 months after intervention (both *P* < 0.001; Table 3). The mean SF-36 score of the patients was significantly higher in the observation group than in the control group (89.96 ± 5.62 vs. 79.05 ± 6.43, respectively; *P* < 0.001). Within both groups, SF-36 scores improved significantly from baseline to 3 months after intervention (both *P* < 0.001; Table 3).

## Discussion

Understanding TFFs management is vital because of its significant impact on the older adult population, particularly on those with frailty. The incidence of TFFs is rising globally, attributed to age-related factors such as osteoporosis and decreased musculoskeletal strength, which predispose older adults to falls and fractures (18). These fractures often lead to severe complications, including mobility impairment and increased mortality rates, thereby imposing a considerable economic burden on healthcare systems (1, 18). The management of TFFs in frail older adults poses unique challenges, as conventional treatment options may not yield optimal functional outcomes, highlighting the necessity for targeted interventions to enhance recovery and improve quality of life in this vulnerable demographic (19). This study investigated the effects of multicomponent exercise interventions on recovery outcomes in older adults with TFFs and frailty, aiming to fill the gaps in existing treatment protocols. Previous research has demonstrated that exercise significantly benefits functional recovery and overall well-being among older individuals; however, frail individuals often remain underrepresented in clinical studies (20, 21).

Multicomponent exercise interventions can promote postoperative rehabilitation in older adults with TFFs and frailty. These patients face not only the pain and functional limitations caused by the fracture, but also an overall decline in physical function, which makes the rehabilitation process more complex and prolonged (22). As a comprehensive rehabilitation method, multicomponent exercise interventions have gradually shown unique advantages in the rehabilitation care of older adults in recent years (23, 24). This study implemented multicomponent exercise interventions for older adults with TFFs and frailty after surgery, and the rehabilitation outcomes in the intervention group, as measured by MBI and FIM scores, were higher than those in the control group, while the CFS scores were lower than those in the control group. The mechanisms promoting patient rehabilitation may include the following: first, strength training enhances muscle strength in older adults and improves joint stability (10); second, balance training improves patients' balance abilities and reduces the risk of falls (25); third, joint mobility training increases joint range of motion and prevents joint stiffness (26); fourth, aerobic exercise improves cardiopulmonary function and enhances overall physical fitness (20). These mechanisms work together to promote comprehensive recovery of physical function in older adults with TFFs. In addition, adjunctive strategies that directly promote fracture healing, such as traditional herbal medicine formulations, may act synergistically with exercise-based rehabilitation and merit further investigation in this population.

It should be acknowledged that anatomical or acceptable fracture reduction and stable fixation are the fundamental prerequisites for successful rehabilitation after extracapsular hip fractures; early mobilization and structured exercise can only confer their benefits once these prerequisites have been achieved. In the present study, all patients underwent postoperative radiographic confirmation of satisfactory reduction before the rehabilitation program was initiated, and partial weight bearing was introduced in a standardized, stepwise progression (bed-based exercises → standing and transfer exercises with an ambulation aid → progressive weight bearing) under the supervision of the multidisciplinary team, thereby facilitating functional recovery while protecting the fixation construct. This is consistent with the findings of Hernández–Pascual et al., who demonstrated that, following satisfactory reduction and cephalomedullary nail fixation, early partial weight bearing is associated with improved long-term functional outcomes without an increase in mechanical failure (27).

Multicomponent exercise interventions can improve hip joint function in older adults with frailty associated with TFFs and enhance their quality of life. Trochanteric femoral fractures and their surgical fixation induce characteristic biomechanical changes: fracture displacement and subsequent nail fixation alter the load transfer through the proximal femur and shorten the effective lever arm of the hip abductor musculature, while postoperative disuse accelerates bone loss and atrophy of the peri-articular muscles, collectively impairing gait stability and hip function (28–31). Targeted strengthening and mobility exercises counteract these changes by restoring abductor strength, improving load distribution, and promoting callus remodeling under appropriately graded loading. The results of this study showed that the HHS and SF-36 quality of life scores after the intervention in the observation group were higher than those in the control group. Multicomponent exercise interventions comprehensively enhance patients' physical function by combining various forms of exercise, such as breathing training, resistance training, joint mobility exercises, standing exercise, walking practice, and balance training. Breathing training enhances cardiopulmonary function, improves overall physical fitness, and provides a better physiological basis for the healing and rehabilitation of fracture sites (20). Resistance training helps to increase muscle strength and endurance, improves local stability, and reduces the risk of re-injury at the fracture site (32). Balance training and joint mobility exercises can enhance the range of motion of the hip, knee, and ankle joints, reduce muscle stiffness and pain, while improving patients' balance ability and reducing the risk of falls, assisting patients in performing daily activities independently (33). Standing training improves weight-bearing on the affected limb, single-limb standing time, knee extensor strength, and balance function in patients with hip fractures (34). These targeted training measures collectively promote the recovery of hip joint function, resulting in better performance in HHS scores in the observation group. Meanwhile, aerobic exercise enhances patients' cardiopulmonary function and increases physical endurance and resistance; as physical functions improve, patients' confidence and self-esteem also increase, leading to an improved quality of life (35, 36). It should be noted that the HHS was originally developed to evaluate outcomes after hip arthroplasty and places considerable emphasis on pain and range of motion; it may therefore not fully capture functional recovery, mobility, gait performance, or independence in this predominantly elderly fracture population. Fracture-specific and geriatric functional instruments, such as the FIM—which was pre-specified as a primary outcome in the present study—provide a more comprehensive assessment of postoperative recovery, and future studies in this population should prioritize such measures.

The strengths of this study include the focus on an important and growing geriatric rehabilitation population, the use of multiple validated outcome measures covering activities of daily living, functional independence, hip function, frailty, and quality of life, the prospective documentation of intervention adherence and adverse events, and the structured, protocol-based description of the multicomponent intervention, which supports its potential clinical applicability.

The limitations of this study should be carefully considered. First, the relatively small sample size and non-randomized design may restrict the generalizability of the findings, as the results may not accurately reflect the broader population of older adults with TFFs. The retrospective, non-randomized design inherently carries risks of selection bias and confounding that cannot be completely eliminated. Selection bias: We acknowledge that patients in the observation group (who received multicomponent exercise intervention) may have differed from the control group in ways not captured by our baseline variables (e.g., motivation, family support, socioeconomic status). Potential confounding factors: We now explicitly list potential confounders that were not measured or adjusted for, including: pre-injury functional status, cognitive function, nutritional status, medication use, living situation and social support. Information bias: We acknowledge the potential for inconsistent assessment across different evaluators, although all assessors were trained on standardized protocols. Second, the absence of a multicenter approach may introduce biases related to a single geographic location or limited institutional practices, potentially affecting the outcomes observed. Most importantly, because the two groups were treated in different periods (2023 vs. 2024) under a quasi-experimental, non-concurrent design, historical confounding cannot be excluded. Staff changes, seasonal differences in patient admissions, and concurrent hospital-wide quality improvement initiatives may have differed between the two periods and may have contributed partly to the observed effects. Although the baseline characteristics were comparable between the groups (Table 1), the intervention followed a standardized written protocol, and all assessors were trained on the same standardized instruments, these measures cannot fully replace randomization or statistical adjustment such as propensity score matching (37). Future randomized controlled trials with propensity-based adjustment—and full reporting of effect sizes and confidence intervals—are warranted to confirm our findings. Third, the follow-up period was limited to 3 months, which may not capture longer-term outcomes such as falls, re-hospitalization, mortality, and sustained functional recovery. Previous studies have shown that the benefits of exercise interventions may diminish after the intervention ends. Future studies with longer follow-up periods (6–12 months) are needed to evaluate the sustainability of our findings. These limitations highlight the need for further research with randomized controlled trials and more diverse populations and multicenter collaborations to validate and extend the findings of this study. In addition, although all patients underwent radiographic confirmation of satisfactory reduction before rehabilitation, quantitative reduction-quality parameters (e.g., reduction grade, tip-apex distance, neck-shaft angle restoration, and the Cleveland zone of the lag screw) were not included in the between-group analysis. Because these parameters are well-recognized predictors of fixation failure, their unmeasured imbalance between the historical cohorts represents a potential source of bias, and future studies should formally incorporate these variables.

In conclusion, the findings from this study provide preliminary evidence that multicomponent exercise interventions may help enhance rehabilitation outcomes, reduce frailty, and improve the quality of life in older adults following TFF. Given the quasi-experimental, non-randomized design with historical (non-concurrent) controls, the observed differences should be interpreted as associations rather than definitive causal effects. These findings suggest that tailored exercise programs may be a promising adjunct to standard postoperative care for this vulnerable population; however, integration into routine care should await confirmation in randomized controlled trials or appropriately adjusted prospective studies. Future studies should explore the scalability of these interventions across diverse settings and populations to refine and validate the therapeutic strategies identified in this study.

## Data Availability

The original contributions presented in the study are included in the article/Supplementary Material, further inquiries can be directed to the corresponding author.
